# Nm23H1 mediates tumor invasion in esophageal squamous cell carcinoma by regulation of CLDN1 through the AKT signaling

**DOI:** 10.1038/oncsis.2016.46

**Published:** 2016-07-04

**Authors:** K-T Kuo, C-L Chen, T-Y Chou, C-T Yeh, W-H Lee, L-S Wang

**Affiliations:** 1 Division of Thoracic Surgery, Department of Surgery, Shuang Ho Hospital, Taipei Medical University, Taipei, Taiwan; 2 Division of Thoracic Surgery, Department of Surgery, School of Medicine, College of Medicine, Taipei Medical University, Taipei, Taiwan; 3 Translational Research Laboratory, Shuang Ho Hospital, Taipei Medical University, Taipei, Taiwan; 4 Department of Pathology and Laboratory Medicine, Taipei Veterans General Hospital, Taipei, Taiwan; 5 Institute of Clinical Medicine, National Yang-Ming University, Taipei, Taiwan; 6 Department of Pathology, Shuang Ho Hospital, Taipei Medical University, Taipei, Taiwan; 7 Graduate Institute of Clinical Medicine, College of Medicine, Taipei Medical University, Taipei, Taiwan

## Abstract

Esophageal cancer is a lethal malignancy worldwide. Previously, low expression of metastasis suppressor Nm23H1 and tight junction (TJ) protein claudin-1 (CLDN1) have been known to correlate with poor prognosis in esophageal squamous cell carcinoma (ESCC). However, the molecular interaction between them has not been clarified. In the present study, we first examined the expression of Nm23H1 and CLDN1 in 74 surgical ESCC samples by immunohistochemistry (IHC) to verify their clinicopathologic significance. The biologic effects of *Nm23H1* gene silencing or overexpression in ESCC cell lines were then studied by migration and invasion studies, and its regulation on CLDN1 expression was also investigated by western blot analysis. Moreover, the expression of Nm23H1 and CLDN1 at the same invasion front of ESCC tumors was verified by immunofluorescence. The results showed a significantly positive correlation between the expression of Nm23H1 and CLDN1 (*γ*=0.296, *P*=0.011) in surgical specimens, especially for the 34 tumors with lymph-node metastasis (*γ*=0.455, *P*=0.007). In ESCC cell lines, silencing of Nm23H1 expression markedly enhanced cell invasiveness, accompanied by increased Akt phosphorylation and decreased CLDN1 expression. Conversely, Nm23H1-expressed transfectants exhibited reduced invasiveness, decreased Akt phosphorylation and correspondingly increased CLDN1 expression. Regain of CLDN1 expression in ESCC cells significantly suppressed invasiveness, but did not influence the Akt phosphorylation. Moreover, treating Nm23H1-depleted cells with the AKT inhibitor MK2206 recovered CLDN1 expression, and diminished the invasiveness of ESCC cells. Finally, decreased expressions of both CLDN1 and E-cadherin were observed at the invasive front of the Nm23H1-negative tumors. Overall, our current study documented that reduced Nm23H1 expression activates the AKT signaling pathway, results in diminished CLDN1 expression and potentiates invasiveness of ESCC cells. Enhancement of Nm23H1 expression, inhibition of the AKT signaling pathway, or combined, might be a potential treatment strategy in selective ESCC patients.

## Introduction

Esophageal cancer is a lethal malignancy worldwide. Most esophageal cancers in Asian countries are esophageal squamous cell carcinomas (ESCC). In the past two decades, the introduction of preoperative chemoradiation has enabled more complete surgical resections of esophageal cancer^1, 2, 3^ but the 5-year survival of this malignancy remains below 20% until now.^4^ Late diagnosis, early tumor metastasis and lack of effective chemotherapy or target therapy are considered as the major obstacles to satisfactory treatment outcomes of esophageal cancer. Most ESCC patients die of extensively local tumor invasion or widespread metastasis. Therefore, identifying factors leading to tumor invasion may be helpful in developing new treatment strategies for this deadly disease.

The *Nm23H1* gene was first identified in 1988 as a tumor metastasis suppressor that was expressed in low quantities in highly metastatic rodent tumors as compared with non-metastatic tumors.^5^ In humans, a decrease in Nm23H1 expression has been found to be associated with aggressive behavior in many malignant tumors, including melanoma, breast, colon, gastric, lung and oral carcinoma.^6, 7, 8, 9^ In our previous studies, we have demonstrated that decreased Nm23H1 expression was associated with poor survival in both ESCC and early-stage oral squamous cell carcinoma, despite that the clinicopathololgic significances of Nm23H1 expression were different in these two tumors.^10, 11^ Biologically, *Nm23H1* encodes a nucleoside diphosphate kinase that has been demonstrated to be involved in several cellular activities of cancers including cell proliferation, differentiation, motility and cell–cell contact.^12, 13, 14^ However, the connection between dysregulation of Nm23H1 and tumor invasion has not been well established.

Disruption of the cell-to-cell junction with concomitant changes in the expression of junctional proteins is a hallmark of cancer invasion and metastasis. Cell-to-cell adhesiveness within the epithelial cell layer is mainly composed of adherens junctions and tight junctions (TJs). Claudins are the major constituents of epithelial TJs and largely participate in forming paracellular barriers as well as making intercellular connections. Their expression and clinicopathologic significance usually vary and depend on cell types and tissues.^15, 16^ Decreased expression of claudin-1 (CLDN1) has been reported to positively correlate with poor prognosis in colon cancer^17^ and lung adenocarcinoma,^18^ as well as tumor recurrence in breast cancer and ESCC.^19, 20^ On the other hand, several *in vitro* studies have shown that increased expression of CLDN1 was associated with increased invasion and metastatic behavior in colon cancer, hepatoma and oral cancer.^21, 22, 23^ Given these controversial data, the accurate biological role of CLDN1 in ESCC remains unclear. Moreover, although both Nm23H1 and CLDN1 have been linked to tumor progression in the literature, whether there is any interaction between them has not been reported previously. In the present study, we investigated the biological relationship between Nm23H1 and CLDN1, and clarified their roles in tumor invasion of ESCC cells.

## Results

### Decreased Nm23H1 expression correlates with lymph-node metastasis and decreased CLDN1 expression in ESCC surgical specimens

**Figure 1:**
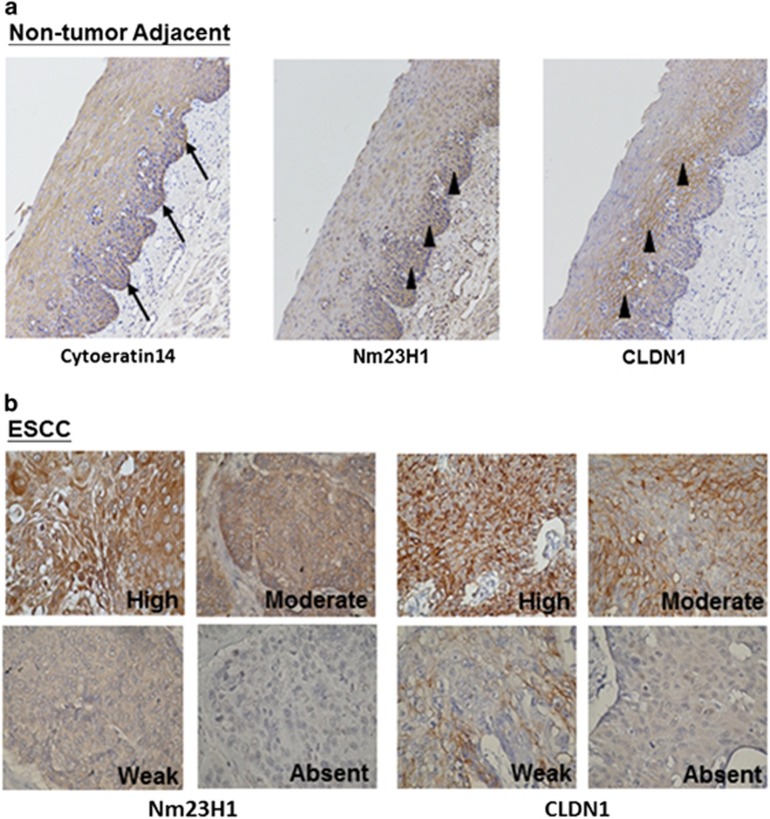
IHC expression of Nm23H1 and CLDN1 in human adjacent non-tumor mucosa and ESCC. (**a**) Nm23H1 and CLDN1 (arrowheads) in tumor-adjacent normal esophageal tissues counterstaining with hematoxylin. The basal layer was highlighted by cytokeratin 14 (arrows). (**b**) Nm23H1 and CLDN1 in ESCC with high, moderate, weak and absent staining. Original magnification, × 100 in (**a**) and × 200 in (**b**).

The expressions of Nm23H1 and CLDN1 in non-tumor epithelial mucosa and tumor cells were comparatively shown in representative surgical specimens in Figure 1. The basal layer of mucosa was highlighted by the specific marker cytokeratin 14. A positive immunoreactivity of Nm23H1 was detected in all of the non-tumor mucosal epithelium of the esophagus (*n*=10), which expressed exclusively in the basal layer, and mildly decreased its intensity toward the upper layers (Figure 1a). Similar to Nm23H1, CLDN1 was predominantly expressed close to the basal layer of the non-tumor mucosal epithelium with membrane-associated localization (Figure 1a). Representative photos of ESCC with different expression intensity for Nm23H1 and CLDN1 were shown in Figure 1b. As illustrated in Table 1, negative or reduced expression of Nm23H1 was found in 30 (40.5%) of the 74 tumor samples. There was a significantly higher percentage of negative or reduced Nm23H1 expression in the metastatic group (*n*=34) than in the non-metastatic group (*n*=40) (61.8 vs 22.5%, *P*<0.001). However, such relationship did not exist in CLDN1 (44.1 vs 35%, *P*>0.05). Meanwhile, the expressions of Nm23H1 and CLDN1 were concomitantly decreased in 38.2% (13 of 34) of the metastatic group, as compared with 10% (4 of 40) of the non-metastatic group. Notably, negative or reduced Nm23H1 expression was significantly correlated with negative or reduced CLDN1 expression (*γ*=0.296, *P*=0.011), especially for the 34 tumors with lymph-node metastasis (*γ*=0.455, *P*=0.007). These results suggested a positive correlation between Nm23H1 and CLDN1 expression in ESCC tumors, and negative or reduced Nm23H1 expression was an indicator for lymph-node metastasis.

### Silencing Nm23H1 expression in the CE48T and the CETE2 cells by shRNA increases cell migration and invasion accompanied by changes in junctional proteins

**Figure 2:**
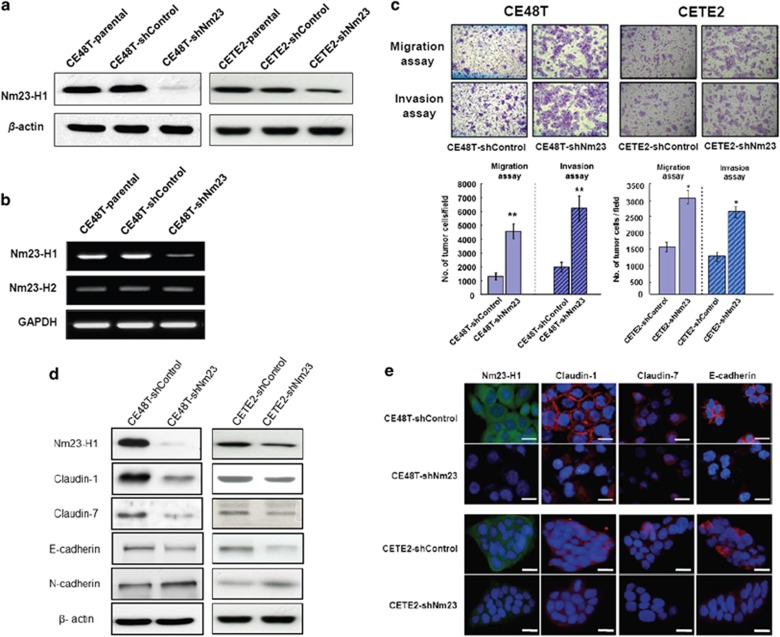
Silencing of Nm23H1 expression in the CE48T and the CETE2 cells increases cell migration and invasion accompanied by changes in junctional proteins. (**a**) Reduced expression of Nm23H1 in the CE48T and the CETE2 cells was achieved. (**b**) RT–PCR analysis revealed that the mRNA expression level of Nm23H2 in the CE48T-shNm23 cells was unaffected. (**c**) Representative photos of migration and invasion assays. Original magnification, × 100. The increased folds of migration and invasion were larger in the CE48T cells. The bar graphs presented the mean values obtained from three independent determinations (**P<0.01; *P<0.05). (**d**) Western blot analysis for Nm23H1, CLDN1, CLDN7, E-cadherin, N-cadherin and β-actin. (**e**) Nm23H1 (green), CLDN1 (red), CLDN7 (red) and E-cadherin (red) in the ESCC-shNm23 and the ESCC-shControl cells were detected by immunofluorescence staining. The cells were also counterstained with DAPI (blue) to localize the nucleus. Scale bar, 20 μm.

To further investigate our initial observations, we employed four different sequences (Supplementary Table 1) of *Nm23H1*-specific shRNA-mediated RNA interference to silence *Nm23H1* expression in the CE48T and the CETE2 cells. Stable clonal CE48T and CETE2 cells after transfection of Nm23-752 and Nm23-750, respectively, were obtained. As shown in Figures 2a and b, highly reduced *Nm23H1* mRNA and protein levels were obtained without changing the expression of Nm23H2 isoform in the CE48T cells. Notably, the suppression of Nm23H1 expression in the CETE2 cells was less prominent than that in the CE48T cells. The Nm23H1 protein level in the CE48T-shNm23 cells was reduced by nearly 90% as compared with the parental CE48T and the CE48T-shControl levels, whereas it was reduced by around 60% in the CETE2-shNm23 cells.

To assess the effects of Nm23H1 on the invasiveness of ESCC cells, a comparative analysis of cell motility between the ESCC-shNm23 and the ESCC-shControl cells was performed by Transwell migration and Matrigel invasion assays. As shown in Figure 2c, substantially larger numbers of the ESCC-shNm23 cells migrated and invaded across the membrane than the ESCC-shControl cells did (*P*<0.01 or *P*<0.05). Not surprisingly, the degree of difference between these two kinds of clones was more remarkable in the CE48T cells, which gained more suppression of Nm23H1 expression after gene silencing.

We further examined the expression of TJ proteins (CLDN1, CLDN7) and cell adhesion proteins (E-cadherin and N-cadherin). As shown in Figure 2d, the expressions of CLDN1, CLDN7 and E-cadherin were significantly decreased in the ESCC-shNm23 cells. A cadherin switch phenomenon with a decreased E-cadherin and an inversely increased N-cadherin was also observed in the ESCC-shNm23 cells. These results documented that silencing *Nm23H1* expression would negatively regulate the expression of CLDN1, CLDN7 and resulted in the cadherin switch.

Furthermore, we validated the effect of *Nm23H1* silencing on the changed expression of CLDN1, CLDN7 and E-cadherin by immunofluorescence analysis. As shown in Figure 2e, Nm23H1 was mainly expressed in the cytoplasm of the ESCC-shControl cells, whereas CLDN1 and E-cadherin were predominantly expressed at cell–cell contact sites. In addition to decreased CLDN1 expression after *Nm23H1* silencing, the localization of the CLDN1 protein also moved from cell–cell contact sites to the cytoplasm and nucleus of the ESCC-shNm23 cells. Thus, our data showed that a deficiency in Nm23H1 expression in ESCC cells might diminish intercellular adhesion and therefore enhance cell invasion.

### Nm23H1-GFP expression in the CE48T and the CETE2 cells suppresses cell migration, invasion and significantly increased CLDN1 expression

**Figure 3:**
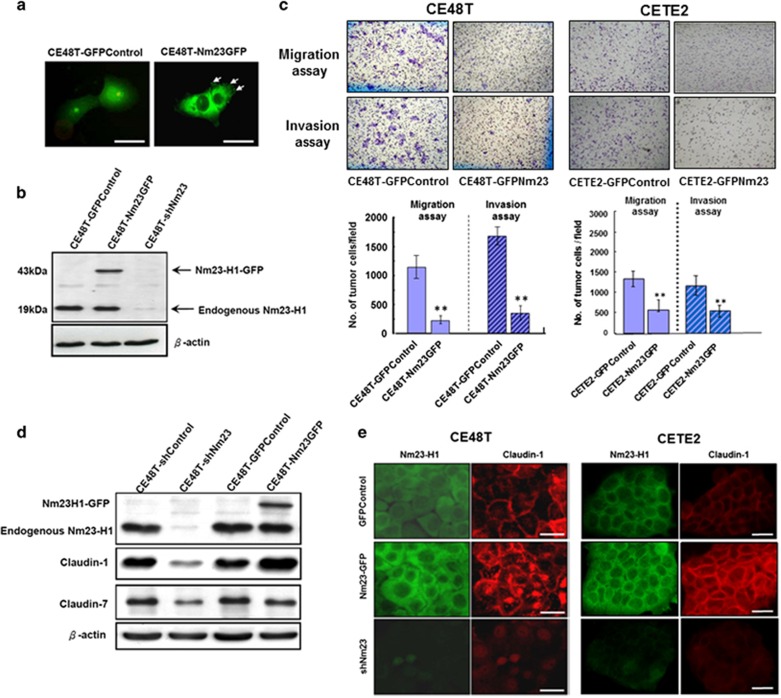
Overexpression of Nm23H1 suppresses cell migration, invasion and increases CLDN1 expression of ESCC cells. (**a**) The CE48T cells were transiently transfected with either GFP plasmids (control) or Nm23H1-GFP plasmids. High magnifications showed a diffuse cytosolic GFP signal in the GFP vector-transfected cells, whereas the Nm23H1-GFP signal can be clearly visualized at the cytoplasm area as well as in the cell trailing edge (arrows). Scale bar, 20 μm. (**b**) The CE48T cells were transiently transfected with the expression plasmids of GFP-vector (GFPControl), Nm23H1-GFP (Nm23GFP) and Nm23-shRNA (shNm23). Expression of Nm23H1 was detected at the predicted molecular mass of 43 kDa (Nm23H1-GFP) or 19 kDa (endogenous Nm23H1). (**c**) Representative photos of migration and invasion assays of the ESCC-GFPControl and the ESCC-Nm23GFP cells. Original magnification, × 100. The bar graphs presented the mean values obtained from three independent determinations (**P<0.01). (**d**) Western blot analysis for Nm23H1, CLDN1, CLDN7 and β-actin in the shRNA-control, the Nm23H1-silencing, the GFP-control and the Nm23H1-GFP clones of the CE48T cells. (**e**) High magnifications showed the expression and localization of Nm23H1 (green) and CLDN1 (red) in the ESCC-GFPControl, the ESCC-Nm23GFP and the Nm23H1-silencing cells. Scale bar, 20 μm.

In addition to the *Nm23H1*-silencing studies, we also enhanced Nm23H1 expression by transfecting the Nm23H1-GFP vector into the CE48T and the CETE2 cells. The subcellular localization of the Nm23H1-GFP was examined using fluorescent microscopy. The Nm23H1-GFP signal could be clearly visualized in the cytoplasm and the cell trailing edge (arrows, Figure 3a), but the GFP signal was mainly distributed in the nucleus and cytoplasm of the GFP-Control cells (Figure 3a). Nm23H1 tagging with GFP was shown as a 43-kDa protein on a western blotted membrane that was recognized by anti-Nm23H1 antibodies. The endogenous Nm23H1 expression in the GFP-Control, the Nm23H1-GFP and the *Nm23H1*-silencing transfectants was also examined for comparison (Figure 3b). We then investigated whether the Nm23H1-GFP expression could suppress cell migration and invasion. As shown in Figure 3c, the ESCC-Nm23GFP cells exhibited a considerably lower ability to migrate and invade than the ESCC-GFPControl cells did (Figure 3c). These results strongly supported that increased Nm23H1 expression might repress the invasiveness of ESCC cells.

To further study the effects of exogenous Nm23H1 expression on CLDN1 and CLDN7 expression, western blot was performed to evaluate the expression of these three proteins in the shRNA-control, the Nm23H1-silencing, the GFP-control, and the Nm23H1-GFP clones of the CE48T cells, respectively (Figure 3d). Nm23H1-GFP expression was positively correlated with CLDN1 expression, but not CLDN7. In immunofluorescent analysis, a considerably stronger intensity of CLDN1 was detected at the cell cytoplasm and cell–cell contact membrane of the Nm23H1-GFP cells, as compared with the GFP-control or the *Nm23H1*-silencing cells (Figure 3e).

### Nm23H1 expression is associated with decreased Akt phosphorylation and enhanced CLDN1 expression

**Figure 4:**
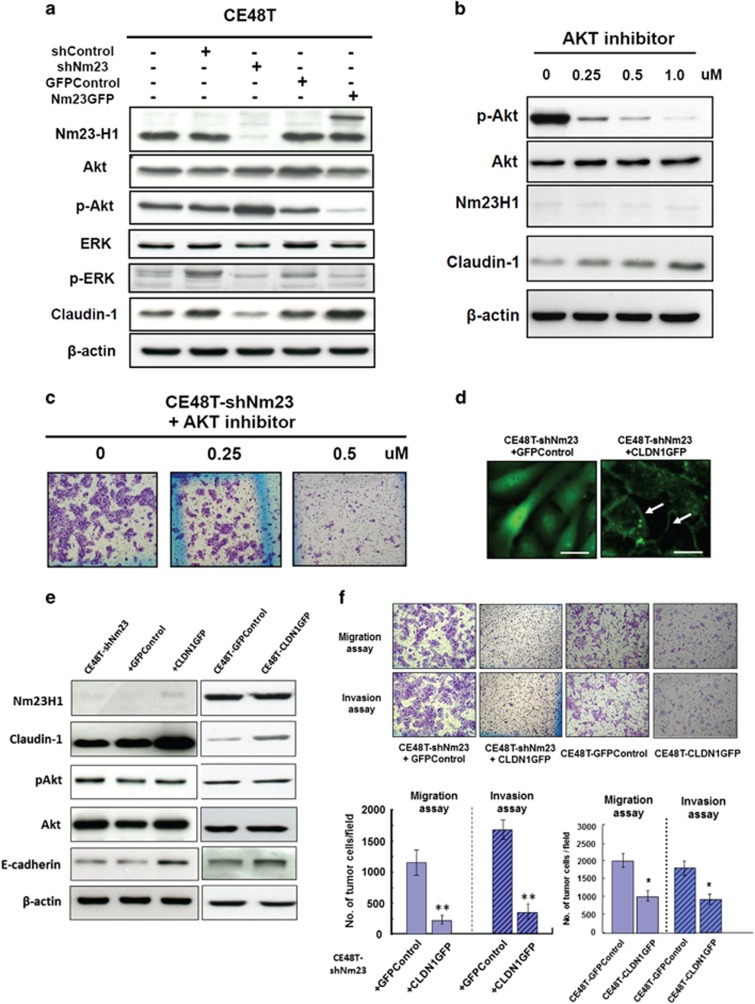
Nm23H1 expression regulates Akt phosphorylation and CLDN1 expression in the CE48T cells, and expression of CLDN1GFP in the CE48T cells suppresses cell migration and invasion independently of Nm23H1. (**a**) Western blot analyses of Nm23H1, Akt, p-Akt, ERK, p-ERK, CLDN1 and β-actin in the shRNA-control (shRNA vector), GFP control (GFP vector), Nm23H1-silencing (shNm23), Nm23H1-GFP and parental CE48T cells were shown. (**b**) Western blot analysis of p-Akt, Akt, Nm23H1, CLDN1 and β-actin in the CE48T-shNm23 cells treated with the AKT inhibitor (MK2206) at the indicated concentration. (**c**) Effects of MK2206 on migration of the CE48T-shNm23 cells were assessed by Transwell migration assay. Original magnification, × 100. (**d**) CLDN1GFP was predominantly localized to the membrane of the Nm23H1-silencing cells at cell–cell contact points (arrows), a pattern that was morphologically similar to the TJ strand network *in situ*, whereas a diffuse cytosolic GFP signal was found in the GFP vector-transfected Nm23H1-silencing cells. (**e**) Western blot analysis for the protein level of CLDN1, pAkt, Akt, E-cadherin and β-actin in the CE48T-shNm23 cells and the CE48T cells with or without CLDN1GFP expression. (**f**) The migration and invasion assays of the representative results were shown. The bar graphs presented the mean values obtained from three independent determinations (**P<0.01; *P<0.05).

Two signaling molecules, including Akt and extracellular signal-regulated kinase (ERK) 1/2, were examined by western blot analysis. As shown in Figure 4a, no significant change in Akt and ERK expression was found in either the CE48T-shNm23 or the CE48T-Nm23GFP cells. However, a higher level of the phosphorylated (p)Akt was found in the CE48T-shNm23 cells, and Akt phosphorylation was again repressed in the CE48T-Nm23GFP cells as compared with the control cells. In contrast, comparing with the parental cells, no significant change in the phosphorylated (p)ERK was detected in either the CE48T-shNm23 or the CE48T-Nm23GFP cells. Meanwhile, the expression of CLDN1 was opposite to the degree of pAkt. These results suggested that the suppression of invasiveness by Nm23H1 in ESCC cells may act through inhibition of Akt phosphorylation, which further contribute to changes in cell-to-cell adhesion, cell migration and invasion relating to CLDN1. To further confirm this speculation, we treated the CE48T-shNm23 cells with MK2206, which is a specific Akt inhibitor, and evaluated the drug effect on expression of CLDN1 and cell migration or invasion. As shown in Figure 4b, CLDN1 expression was recovered in a dose-dependent manner in the CE48T-shNm23 cells after the treatment of MK2206. Moreover, MK2206 markedly reduced the migration and invasion of the CE48T-shNm23 cells (Figure 4c). This result further suggested that CLDN1 is likely to be a downstream gene of the AKT signaling mediated by Nm23H1.

The effects of suppressing Nm23H1 on Akt phosphorylation and CLDN1 expression were further tested in two other ESCC cell lines (CE146T and CETE2) (see Supplementary Figure S1). Consistent with the results of the CE48T cells, silencing of Nm23H1 expression by shRNA caused increased pAkt levels and reduced CLDN1 expression in both the CE146T and the CETE2 cells.

### Expression of CLDN1GFP in the CE48T cells suppresses cell migration and invasion independently of Nm23H1 expression

To ascertain whether the significant increase of cell migration and invasion in the CE48T-shNm23 cells was mediated by reduced endogenous CLDN1, we transiently transfected the CLDN1GFP vectors or the GFP-control vectors into the CE48T-shNm23 cells and the parental CE48T cells for investigation. As shown in Figure 4d, CLDN1GFP predominantly localized at cell–cell contact sites of CE48T-shNm23+CLDN1GFP cells. By contrast, the CE48T-shNm23+GFPControl cells showed only cytoplasmic GFP fluorescence instead of fluorescence at cell–cell contact sites. The left columns of Figure 4e confirmed that high CLDN1 expression was obtained in CE48T-shNm23+CLDN1GFP cells. However, no significant difference in pAkt levels was found between the CE48T-shNm23+CLDN1GFP cells and the CE48T-shNm23+GFPControl cells, although E-cadherin expression was slightly stronger in the CE48T-shNm23+CLDN1GFP cells. This indicated that CLDN1 did not have an upstream role in regulating Akt phosphorylation but may be associated with E-cadherin expression. Similar findings were found in the CE48T-GFPControl cells and the CE48T-CLDN1GFP cells (right columns of Figure 4e). To further address the impact of re-expression of CLDN1 on invasiveness, the migration and invasion of these four clones were further assessed. As shown in Figure 4f, the cell migration and invasion of the CE48T-shNm23+CLDN1GFP cells were significantly less than the CE48T-shNm23+GFPControl cells, and so were the CE48T-CLDN1GFP and the CE48T-GFPControl cells. These results revealed that recovery of CLDN1 expression in ESCC cells could suppress their migration and invasion in a Nm23H1-independent manner.

### Relationship of expressive localizations among Nm23H1, CLDN1 and E-Cadherin at the invasive front of primary ESCC tumors

**Figure 5:**
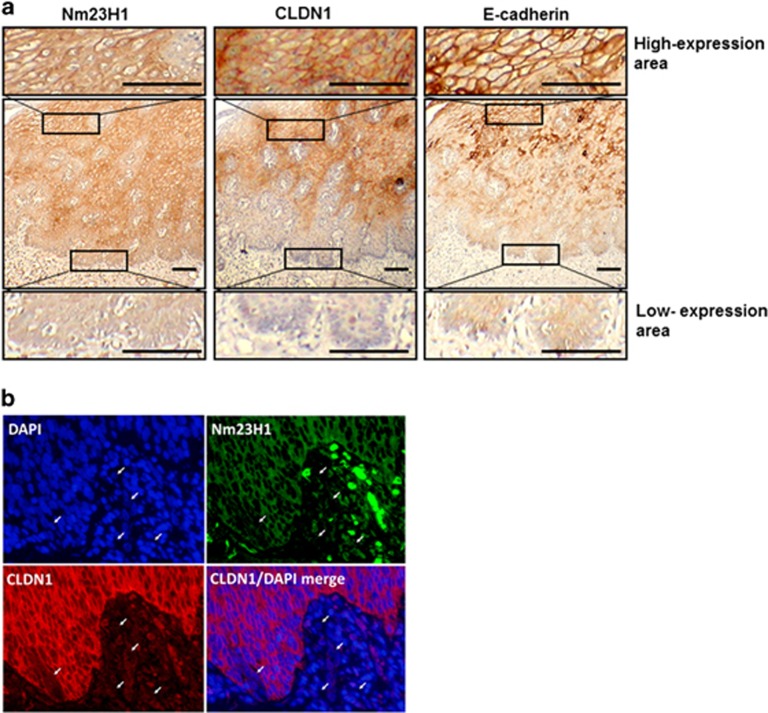
Expression levels of Nm23H1 at the invasive front of the ESCC tumor. (**a**) IHC analysis of expression patterns of Nm23H1, CLDN1 and E-cadherin in sequenced sections was shown. Representative images showed the reduced expression of Nm23H1, CLDN1 and E-cadherin at the invasive front of the ESCC tumor (lower panels) and high expression levels of Nm23H1, CLDN1 and E-cadherin in the main body of ESCC tumor (upper panels). Scale bar, 200 μm. (**b**) Representative double immunofluorescent images showed that several single ESCC cells appeared with losing or reducing Nm23H1 (green) and CLDN1 (red) expression (arrows), respectively. Nuclear DNA was stained with DAPI (blue). Scale bar, 20 μm.

On the basis of the IHC analysis and cell line studies, we speculated that reduction of Nm23H1 and CLDN1 expression would be a common phenomenon in aggressive ESCCs. The results of the immunoreactivity of Nm23H1, CLDN1 and E-cadherin at the invasive front of the same primary ESCC tumors were shown in Figure 5a. At the invasive front (Figure 5a, lowest panels), Nm23H1 and CLDN1 staining was dramatically reduced, whereas E-cadherin showed less intensity in the cellular cytoplasm and lost its cell-membrane localization. However, CLDN1 and E-cadherin were positively stained at the cell–cell contact area and exhibited a honeycomb-like appearance in the highly Nm23H1-expressed area of the same section (Figure 5a, uppermost panels). In addition, duplicate immunofluorescent staining revealed co-localization of Nm23H1 and CLDN1 in the same primary tumor (Figure 5b). At the invasive front of the tumor, single cancer cells appeared to lose or reduce their expressions of both Nm23H1 and CLDN1 (Figure 5b, arrows). This evidence further supported our aforementioned results of ESCC cell experiments.

## Discussion

Nm23H1 protein is the first identified metastasis suppressor with the initially recognized characteristic of its ability to inhibit formation of metastasis without affecting the primary tumor growth *in vivo*. With more and more studies, the anti-metastatic effects of Nm23H1 were gradually unfolded, including its enzymatic activities (nucleotide diphosphate kinase, histidine kinase and 3'-5' exonuclease), protein–protein interactions and regulation of downstream genes.^6, 24^ Our previous studies have shown that the expression of Nm23H1 was associated with better survival in patients with ESCC and oral squamous cell carcinoma. However, unlike oral squamous cell carcinoma, we did not find a correlation between Nm23H1 expression and lymph-node metastasis in that ESCC cohort. In the present study, using another ESCC cohort, we documented that patients with low expression of Nm23H1 were significantly associated with more lymph-node metastasis. As for the explanation of this discrepancy, we considered there are two possible reasons. The first one is that these are two totally different cohorts, with the former one between 1985 and 2000 and the later one between 2009 and 2011. The heterogeneity within these two cohorts *per se* may contribute to different results. The second one is that we used different ways to score the IHC data. In the former study, we applied 20% of stained cancer cells as the cutoff value for positivity. In the later study, we used a multiplying score as the cutoff value for positivity. Such difference may be reflected in the fact that the positive rate of Nm23H1 in former cohort was 39.3%, whereas the positive rate of Nm23H1 in the later cohort was 59.5%.

Cell line studies also demonstrated that modulation of Nm23H1 influences expression of multiple proteins that were accompanied by changes in cell-to-cell adhesion, migration and invasion. These proteins included CLDN1, CLDN7, E-cadherin and N-cadherin, which were all considered components of epithelial–mesenchymal transition. Therefore, we speculated that the anti-metastatic function of Nm23H1 in ESCC cells is closely related to its role in epithelial–mesenchymal transition. Of the proteins influenced by Nm23H1, CLDN1 may have a major role. As shown in Table 1, in ESCC tumors with lymph-node metastasis, the expression of Nm23H1 and CLDN1 significantly correlated to each other, whereas in ESCC tumors without lymph-node metastasis, such correlation did not exist. At the invasive front of the same ESCC tumor, negative expressions of both Nm23H1 and CLDN1 were simultaneously observed (Figure 5). This further highlighted the intimate relationship between these two proteins.

Regarding the regulatory mechanism of CLDN1 by Nm23H1, we found that Nm23H1 was a negative modulator for Akt phosphorylation, which also negatively regulates CLDN1 expression in a dose-dependent manner in the Nm23H1-depleted ESCC cells. By treating the Nm23H1-depleted cells with the Akt inhibitor, we further confirmed that the AKT signaling was required to develop invasiveness in the Nm23H1-depleted cells (Figures 4b and c). Furthermore, we demonstrated that enhanced CLDN1 expression by gene transfection markedly diminished their migration and invasion without changing either Nm23H1 expression or Akt phosphorylation, and was independent of Nm23H1 expression (Figures 4e and f). Taken together, we propose that there is a likely causal relationship between Nm23H1 and CLDN1 in ESCC cells. In fact, apart from Nm23H1, multiple signaling pathways or factors including ERK 1/2, PI3K/AKT, Smad, β-catenin and the Snail family have been implicated as regulators of CLDN1 expression.^22, 25, 26^ However, to the best of our knowledge, our report is the first one that identifies the regulation of CLDN1 expression through the AKT signaling by Nm23H1.

As for other proteins influenced by Nm23H1, we found that E-cadherin and CLDN7 were also involved (Figure 2d). However, the expression of Snail and Twist was not affected by modulation of Nm23H1 (data not shown). Further analysis revealed that expression of E-cadherin seemed parallel to CLDN1 instead of Nm23H1 (Figure 4e), whereas expression of CLDN7 seemed not directly regulated by Nm23H1 (Figure 3d). Previously, it has been reported that silencing of CLDN7 by genetic approaches leads to reduced E-cadherin expression, impairment of homotypic adhesion and increased cell invasion.^27^ Therefore, from our current data, we speculated that E-cadherin and CLDN7 were affected collaterally by Nm23H1, whereas CLDN1 was likely directly regulated by Nm23H1.

Because many patients with esophageal cancer have extensively local invasion and even metastasis at diagnosis,^28^ how to manage tumor aggressiveness remains a challenging task in the treatment of esophageal cancer. Our current study suggested that Nm23H1 may have an important role in tumor invasion in ESCC cells, and therefore can be a useful target for ESCC. Actually, therapeutic strategies aiming to increase Nm23H1 expression in order to reduce cancer metastasis have been proposed in the past decade, and some of them have been applied in clinical settings. Such approaches included medroxyprogesterone acetate,^29, 30^ gene therapy^31^ and lysophosphatidic acid,^32^ and some candidate drug targeting lysophosphatidic acid could even induce tumor dormancy at secondary tumor sites.^33^ Moreover, in our current study, application of the Akt inhibitor MK2206 was able to abrogate the invasiveness of Nm23H1-depleted ESCC cells significantly. Altogether, our data implied that in selective ESCC patients, enhancement of Nm23H1 expression, inhibition of the AKT signaling pathway, or combined, may be a potential treatment strategy.

In conclusion, our current study demonstrated that reducing Nm23H1 in ESCC cells may inactivate the AKT signaling pathway and downregulate CLDN1 expression, thereby increase the invasiveness of ESCC cells.

## Materials and methods

### IHC analysis in tumor and non-tumor mucosal specimens

A total of 74 primary ESCC specimens and the 10 corresponding non-tumor mucosal epithelia were collected from 74 ESCC patients undergoing subtotal esophagectomy between June 2009 and June 2011, after obtaining written informed consents from all the patients. Histopathologically, all tumor samples were confirmed by pathologists to be ESCC. Among these 74 tumor specimens, 34 samples were taken from patients with locoregional lymph-node metastasis (the metastatic group), and the other 40 samples were from patients without lymph-node involvement (the non-metastatic group). The IHC staining was performed as described in our previous report.^10^ Tissue microarrays or tissue slides were incubated with anti-Nm23H1 antibody (sc-343; Santa Cruz Biotechnology, Inc., Dallas, TX, USA), anti-CLDN1 antibody (ab15098; Abcam plc, Cambridge, UK), anti-cytokeratin 14 antibody (ab7800, Abcam plc) and anti-E-cadherin antibody (ab1416; Abcam plc). IHC scoring was assessed by two investigators in a blind manner.

### Grading and scoring of IHC staining

Nm23H1 and CLDN1 staining intensity was evaluated semi-quantitatively and graded for both intensity (absent or weak, 1; moderate, 2; strong, 3) and extent (percentage of positive cells: <10%, 1; 10%–50%, 2; >50%, 3). Intensity and extent scores were multiplied to obtain a composite score of 1–9 for each specimen. Composite scores of 1–4 were defined as having negative or reduced protein expression, and scores of 5–9 were considered as positive expression.

### Esophageal cancer cell lines and cell culture

The human esophageal cancer cell lines, CE48T, CE146T and CETE2 (obtained from the Food Industry Research and Development Institute, Hsinchu, Taiwan), were grown in monolayer cultures and maintained in a high-glucose Dulbecco's modified Eagle's medium (DMEM) supplemented with 10% fetal bovine serum, 100 μg/ml streptomycin, 100 units/ml penicillin G and 3.75 μg/ml sodium bicarbonate at 37 °C in a 5% CO_2_ environment. Cells at the fifth to ninth passages of the exponentially growing state were used for all experiments.

### Gene silencing and gene overexpression

Gene silencing was conducted by short-hairpin RNA. The CE48T and CETE2 cells were maintained in DMEM supplemented with 10% fetal bovine serum, sodium pyruvate (1 mm), HEPES (10 mm) and β-mercaptoethanol (50 μm). Cells were stably transfected with the pRS vector (# TR20003; cell line Vec-shRNA; Origene, Rockville, MD, USA), pRS-short hairpin (sh) GFP (Origene # TR30003; encoding a non-effective 29-mer shGFP cassette as a negative control; cell line GFP-shRNA), or with a combination of specific *Nm23H1*-directed 29-mer oligos (4 *Nm23H1*-specific shRNAs, as shown in Supplementary Table 1). These 29-mer oligos were incorporated in the forward and reverse orientations, separated by a TCAAGAG loop in vector pRS shRNA, by the manufacturer to generate four pRS-based gene-specific human *Nm23H1* shRNA expression vectors (HuSH; Origene). All were independently tested for their ability to downregulate Nm23H1 expression.

As for gene overexpression, the CE48T cells grown on 60-mm dishes were transfected with 8 μg of pCMV6-AC-GFP vector containing the cDNA for full-length human *Nm23H1* or *CLDN1* by Lipofectamine 2000 reagent (Invitrogen, Carlsbad, CA, USA) according to the manufacturer's instructions. The transfected cells were selected by growing the cells in 1 mg/ml of G418 (Invitrogen) in DMEM. The resistant clones formed were picked up and maintained separately with 100 μg/ml G418 and analyzed for protein expression.

### RNA extraction and semi-quantitative reverse-transcriptase PCR

RNA extraction and gene amplification have been described previously.^34^ Briefly, total RNA was extracted using the Trizol solution (Invitrogen), and 1 mg of total RNA was reverse-transcribed using a RevertAid first-strand complementary (c)DNA synthesis kit (Fermentas, Hanover, MD, USA). The cDNA was amplified from 1 μl of reverse-transcriptase products by PCR amplification in the presence of Taq DNA Polymerase Master Mix Red (Ampliqon, Herlev, Denmark) and the DNAEngine Peltier thermal cycler (Bio-Rad, Hercules, CA, USA). The primer sequences, amplification product sizes and annealing temperatures are shown in Supplementary Table 2.

### Protein extraction and western blot analysis

Procedures for protein extraction and western blot have also been described previously.^34^ In brief, ESCC cell lines were seeded onto culture dishes after 24 h of incubation. Cells were washed and lysed by lysis buffer, and cell lysates were then centrifuged at 12 000 r.p.m. at 4 °C for 10 min. Protein concentrations were measured; 20 μg of protein samples was solubilized by boiling in a sample buffer and subjected to SDS-polyacrylamide gel electrophoresis (PAGE), followed by electrotransfer onto a nitrocellulose membrane (Amersham, Buckinghamshire, UK). Blots were blocked for 1 h at room temperature with 2% non-fat dry milk and incubated at 4 °C overnight with the primary antibodies (Supplementary Table 3). The membranes were incubated with correspondingly secondary antibodies. After additional washing, the membranes were developed with enhanced chemiluminescence reagents (PerkinElmer Western Lightning, Boston, MA, USA), and exposed to film in a dark room.

### Migration and invasion assays

Cell migration assays were performed by modifying some previously published protocol.^35^ A 12-mm Transwell with an 8.0-μm-pore polycarbonate membrane insert (Millipore, Carrigtwohill, Ireland) was used in this assay. ESCC cells were harvested and resuspended into DMEM supplemented with 1% fetal calf serum. The upper chamber of the insert was filled with 150 μl of the cells (2 × 10^4^ cells). The lower chamber was filled with a culture medium supplemented with 10% fetal calf serum as the chemoattractant. The plate was incubated in a humidified environment at 37 °C with 5% CO_2_ for 24 h. After incubation, cells were removed from the upper surface of the membrane by wiping with a moist cotton swab. Cells that had migrated through the membrane and adhered to the lower surface of the membrane were fixed with methanol, stained for 3 min with Giemsa stain (Sigma-Aldrich Inc., St Louis, MO, USA), rinsed with distilled water to remove the excess stain unabsorbed by the cells, and counted under a light microscope ( × 100). For the invasion assay, Matrigel was purchased from BD Biosciences (San Jose, CA, USA) and stored at −20 °C. After thawing at 4 °C overnight, the Matrigel was diluted in serum-free DMEM. The diluted Matrigel (1:20 for invasion) was evenly inoculated into the upper chamber of the 8.0-μm Transwell membrane, and was allowed to form into gel at 37 °C. The remaining processes of the Matrigel invasive assay were the same as those for the migration assay.

### Immunofluorescence microscopy

ESCC cells were seeded onto glass coverslips in 6-well plates and incubated overnight. Cells were then fixed in a 4% formaldehyde solution (Electron Microscopy Systems, Hatfield, PA, USA) and permeabilized with Triton X-100 [0.2% (v/v)]. Samples were blocked in PBS containing 1% BSA. Primary antibody incubations (Nm23H1, CLDN1, Claudin-7 (CLDN7) and E-cadherin) at a 1:100 dilution ratio were performed at 37 °C in a humidified atmosphere for 1 h, followed by secondary antibody incubations (1:250). For immunofluorescence detection of the esophageal tissue sections, fixed paraffin-embedded tissue slides were deparaffinized with xylene and rehydrated with different concentrations of ethanol and washed with PBS. The antigen was retrieved by citrate buffer and the slides were blocked with 10% goat serum. The primary antibodies were incubated overnight at 4 °C, and secondary antibodies were incubated at room temperature for 1 h. After incubation, the slides were washed and treated with a mounting medium, and then photographed under an Olympus FSX100 fluorescence microscope (Olympus Optical Co., Ltd, Tokyo, Japan).

### Statistical analysis

Data were expressed as the mean±s.d. of at least three independent experiments. The statistical differences between the two groups were analyzed using the Student's *t-*test. The correlations between two variables were evaluated using the Pearson chi-squared and Fisher's exact tests. Statistical analysis was performed using the Statistical Package of Social Sciences software, version 13.0 (SPSS, Chicago, IL, USA), a 2-sided *P-*value of <0.05 was considered as the significant statistical level.

## Figures and Tables

**Table 1:** Correlation between Nm23H1 and CLDN1 expression in ESCC samples

*CLDN1 expression*	*Nm23H1 expression*			
	*Positive*	*Negative*	γ	P-*value*
All tumors (*N*=74)			0.296	0.011
Positive	32	13		
Negative	12	17		
Tumors with lymph-node metastasis (*N*=34)			0.455	0.007
Positive	11	8		
Negative	2	13		
Tumors without lymph-node metastasis (*N*=40)			0.107	0.512
Positive	21	5		
Negative	10	4		

Abbreviations: CLDN1, claudin-1; ESCC, esophageal squamous cell carcinoma.
