# Supplementary material for: Nm23H1 mediates tumor invasion in esophageal squamous cell carcinoma by regulation of CLDN1 through the AKT signaling
Source: Oncogenesis. 2016 Jul 4;5(7):e239–. doi: 10.1038/oncsis.2016.46 (PMC4972901; doi:10.1038/oncsis.2016.46)

**Nm23H1 mediates tumor invasion in esophageal squamous cell carcinoma by regulation of CLDN1 through the AKT signaling**

Kuang-Tai Kuo^1,4^, Chien-Lung Chen^2^, Teh-Ying Chou^6,7^, Chi-Tai Yeh^2^, Wei-Hwa Lee^3^, Liang-Shun Wang^1,5*^

Supplementary Table 1. Oligo-sequences of 4types of sh-nm23 and the negative shRNA control.

| **Name** | **Oligo sequence (5'→3')** |
| --- | --- |
| Sh-Nm23-749 (TI311749) | CCTATCTCAAGCTGTGATACAGGAACCAT |
| Sh-Nm23-750 (TI311750) | CGTTGACCTGAAGGACCGTCCATTCTTTG |
| Sh-Nm23-751 (TI311751) | AAAGGATTCCGCCTTGTTGGTCTGAAATT |
| Sh-Nm23-752 (TI311752) | CTGAGGAACTGGTAGATTACACGAGCTGT |
| Scramble (TR30013) | GCACTACCAGAGCTAACTCAGATAGTACT |

Supplementary Table 2. Amplification product sizes and annealing temperatures of the PCR sequences.

| **cDNA** | **Forward primer (5'→3')**  **Reverse primer (5'→3')** | **Product**  **sizes (bp)** | **Annealing**  **temperature (°C)** |
| --- | --- | --- | --- |
| Nm23H1 | **F:**GCAGCCGGAGTTCAAACCTAA  **R:**GCTGGGAGGAAGCATTTTAATCA | 685 | 60 |
| Nm23H2 | **F:**CTGAAGAACACCTGAAGCA  **R:**AATCCTGTTGCCTCTAAGTC | 419 | 60 |
| GAPDH | **F:**ACCACAGTCCATGCCATCAC  **R:**TCCACCACCCTGTTGCTGTA | 452 | 52 |

Supplementary Table 3. Primary antibodies used in the study.

| **Specificity** | **Catalog** | **Company** | **Dilution** | **Apply** |
| --- | --- | --- | --- | --- |
| β-actin | ab6276 | Abcam plc., Cambridge, UK | 1:10000 | WB |
| Nm23H1 | sc-343 | Santa Cruz Biotechnology, Inc., Europe | 1:50 | IHC & WB |
| E-cadherin | ab1416 | Abcam plc., Cambridge, UK | 1:50 | IHC & WB |
| N-cadherin | ab12221 | Abcam plc., Cambridge, UK | 1:100 | WB |
| Cytokeratin 14 | ab7800 | Abcam plc., Cambridge, UK | 1:100 | IHC |
| CLDN1 | ab15098 | Abcam plc., Cambridge, UK | 1:150 | IHC & WB |
| CLDN7 | ab27487 | Abcam plc., Cambridge, UK | 1:150 | WB |
| Akt | #9272 | Cell Signaling Technology, MA, USA | 1:2000 | WB |
| p-Akt | #9271 | Cell Signaling Technology, MA, USA | 1:1000 | WB |
| ERK1/2 | LF-MA0178 | Young In Frontier Co. Ltd., Seoul, Korea | 1:2000 | WB |
| p-ERK1/2 | LF-PA0090 | Young In Frontier Co. Ltd., Seoul, Korea | 1:5000 | WB |

Supplementary Figure S1

Silencing Nm23H1 significantly reduced CLDN1 expressions and negatively correlated with the levels of AKT activation in the CE48T, the CE146T, and the CETE2 cell lines.


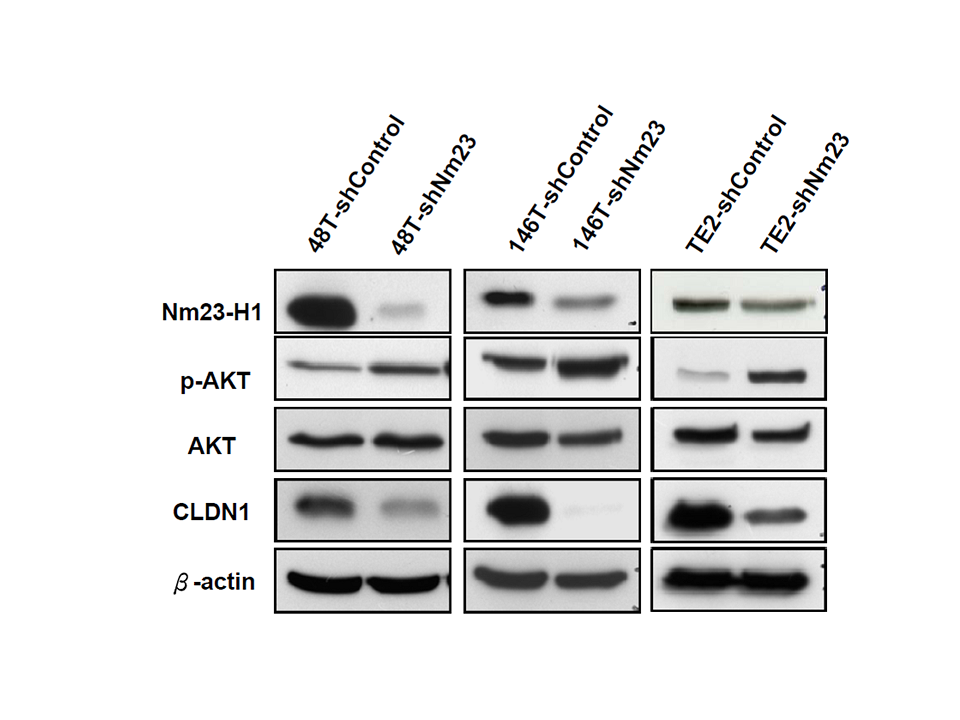

Supplement: Supplementary Information [file oncsis201646x1.doc]
